# Gene Expression Profiling in Wild-Type and PPAR*α*-Null Mice Exposed to Perfluorooctane Sulfonate Reveals PPAR*α*-Independent Effects

**DOI:** 10.1155/2010/794739

**Published:** 2010-09-27

**Authors:** Mitchell B. Rosen, Judith R. Schmid, J. Christopher Corton, Robert D. Zehr, Kaberi P. Das, Barbara D. Abbott, Christopher Lau

**Affiliations:** ^1^Integrated Systems Toxicology Division, Office of Research and Development, National Health and Environmental Effects Research Laboratory, U.S. Environmental Protection Agency, MD 72, Research Triangle Park, NC 27711, USA; ^2^Biostatistics and Bioinformatics Team, Office of Research and Development, National Health and Environmental Effects Research Laboratory, U.S. Environmental Protection Agency, MD 72, Research Triangle Park, NC 27711, USA; ^3^Analytical Chemistry Team, Office of Research and Development, National Health and Environmental Effects Research Laboratory, U.S. Environmental Protection Agency, MD 72, Research Triangle Park, NC 27711, USA; ^4^Toxicology Assessment Division, Office of Research and Development, National Health and Environmental Effects Research Laboratory, U.S. Environmental Protection Agency, MD 72, Research Triangle Park, NC 27711, USA

## Abstract

Perfluorooctane sulfonate (PFOS) is a perfluoroalkyl acid (PFAA) and a persistent environmental contaminant found in the tissues of humans and wildlife. Although blood levels of PFOS have begun to decline, health concerns remain because of the long half-life of PFOS in humans. Like other PFAAs, such as, perfluorooctanoic acid (PFOA), PFOS is an activator of peroxisome proliferator-activated receptor-alpha (PPAR*α*) and exhibits hepatocarcinogenic potential in rodents. PFOS is also a developmental toxicant in rodents where, unlike PFOA, its mode of action is independent of PPAR*α*. Wild-type (WT) and PPAR*α*-null (Null) mice were dosed with 0, 3, or 10 mg/kg/day PFOS for 7 days. Animals were euthanized, livers weighed, and liver samples collected for histology and preparation of total RNA. Gene profiling was conducted using Affymetrix 430_2 microarrays. In WT mice, PFOS induced changes that were characteristic of PPAR*α* transactivation including regulation of genes associated with lipid metabolism, peroxisome biogenesis, proteasome activation, and inflammation. PPAR*α*-independent changes were indicated in both WT and Null mice by altered expression of genes related to lipid metabolism, inflammation, and xenobiotic metabolism. Such results are similar to studies done with PFOA and are consistent with modest activation of the constitutive androstane receptor (CAR), and possibly PPAR*γ* and/or PPAR*β*/*δ*. Unique treatment-related effects were also found in Null mice including altered expression of genes associated with ribosome biogenesis, oxidative phosphorylation, and cholesterol biosynthesis. Of interest was up-regulation of *Cyp7a1*, a gene which is under the control of various transcription regulators. Hence, in addition to its ability to modestly activate PPAR*α*, PFOS induces a variety of PPAR*α*-independent effects as well.

## 1. Introduction

Perfluoroalkyl acids (PFAAs) are stable man-made perfluorinated organic molecules that have been utilized since the 1950s in the manufacture of a variety of industrial and commercial products suchas fire fighting foams, fluoropolymers for the automobile and aerospace industry, paper food packaging, stain-resistant coatings for carpet and fabric, cosmetics, insecticides, lubricants, and nonstick coatings for cookware. One such PFAA, perfluorooctane sulfonate (PFOS), was identified nearly a decade ago as a persistent organic pollutant which could also be found in the tissues of wildlife throughout the globe [2]. Since that time, a number of perfluorinated sulfonic and carboxylic acids of varying chain length have been shown to be persistent and ubiquitous environmental contaminants. Some of these compounds are also commonly identified in the tissues of humans and wildlife with the 8-carbon PFAAs, PFOS and perfluorooctanoic acid (PFOA), being the most frequently reported in biomonitoring studies (for reviews, see [3, 4]). In recent years, blood levels of PFOS and PFOA have gradually begun to decline in the general population [5, 6]. This is due in part to a production phase out of PFOS by its principal U.S. manufacturer as well as a commitment by key manufacturers of perfluorinated chemicals to reduce the product content and emissions of PFOA, and related chemistries, under the EPA 2010/2015 PFOA Stewardship Program (http://www.epa.gov/oppt/pfoa/pubs/stewardship/index.html). Nevertheless, certain PFAAs are likely to remain of concern for years to come due to their environmental persistence and long biological-half lives [7]. 

PFOS and PFOA are associated with toxicity in laboratory animals at blood levels that are approximately 2-3 orders of magnitude above those normally observed in humans. This includes hepatomegaly and liver tumors in rats and mice as well as pancreatic and testicular tumors in rats (for review see [4]). Teratogenic activity has also been observed in rats and mice, however, such findings have been limited to maternally toxic doses of PFOS [8], whereas, both PFOS and PFOA have been shown to alter growth and viability of rodent neonates at lower doses [4]. Recent epidemiologic data suggests that typical exposures to these compounds may alter fetal growth and fertility in humans [9–13]. These studies, however, lack consistency with regard to either compound activity or measured end point; therefore, alternative explanations for such findings have been suggested [14]. Moreover, a recent study of individuals exposed to PFOA in drinking water at levels that were approximately two orders of magnitude higher than the general population did not show an effect on average birth weight or the incidence of low birth weight infants [15]. 

The mode of action related to PFAA toxicity in rodents is not fully understood. As a class of chemicals, PFAAs activate peroxisome proliferator-activated receptor alpha (PPAR*α*) [16–18], and chronic activation of this nuclear receptor is thought to be responsible for the liver enlargement and hepatic tumor induction found in laboratory animals [19]. However, activation of PPAR*α* is not thought to be a relevant mode of action for hepatic tumor formation in humans [20–25], although this assumption has been challenged recently [26]. This does not, however, rule out the possibility that certain PFAAs could have an adverse effect on development since activation of PPAR*α* has been shown to play a role in PFOA-induced neonatal loss in mice [27]. In addition, PPAR*α*-independent modes of action are also likely for various PFAAs. Unlike prototypical activators of PPAR*α*, such as, the fibrate class of pharmaceuticals, PFOA can induce fatty liver in wild-type mice [28]. PFOA can also induce hepatomegaly in PPAR*α*-null mice [27, 29, 30] and is capable of activating the constitutive androstane receptor (CAR) [31–33]. Moreover, PFOS can induce neonatal toxicity in the PPAR*α*-null mouse [34]. 

 In the current study, we used global gene expression profiling to assess the transcriptional changes induced by PFOS in the liver of wild-type and PPAR*α*-null mice. The data were compared to results previously published by our group for PFOA and Wy-14,643, a commonly used agonist of PPAR*α* [1]. Our goal was to identify both PPAR*α*-dependent and independent changes induced by PFOS.

## 2. Materials and Methods

### 2.1. Animals and Dosing

Studies were approved by the U.S. EPA ORD/NHEERL Institutional Animal Care and Use Committee. The facilities and procedures used followed the recommendations of the 1996 NRC “Guide for the Care and Use of Laboratory Animals,” the Animal Welfare Act, and the Public Health Service Policy on the Humane Care and Use of Laboratory Animals. 

PPAR*α*-null (Null) mice (129S4/SvJae-*P* *p* *a* *r* *a* ^tm1Gonz^/J, stock no. 003580) and wild-type (WT) mice (129S1/SvlmJ, stock no. 002448) were initially purchased from The Jackson Laboratory (Bar Harbor, ME) and maintained as an inbred colony on the 129/Sv background at the U.S. EPA, Research Triangle Park, NC. Animals were housed 5 per cage and allowed to acclimate for a period of one week prior to the conduct of the study. Food (LabDiet 5P00 Prolab RHM3000, PMI Nutrition International, St. Louis, MO) and municipal tap water were provided *ad libitum*. Animal facilities were controlled for temperature (20–24°C), relative humidity (40%–60%), and kept under a 12 hr light-dark cycle. The experimental design matched that of our previous study [1]. PPAR*α*-null and wild-type male mice at 6–9 months of age were dosed by gavage for 7 consecutive days with either 0, 3, or 10 mg/kg PFOS (potassium salt, catalog no. 77282, Sigma Aldrich, St, Louis, MO) in 0.5% Tween 20. Five biological replicates consisting of individual animals were included in each dose group. Dose levels were based on unpublished data from our laboratory and reflect exposures that produce hepatomegaly in adult mice without inducing overt toxicity. Animals utilized for RT-PCR analysis were taken from a separate set of WT and Null mice. PCR dose groups consisted of 4 animals per group and were treated for seven-days with either 10 mg/kg/day PFOS, 3 mg/kg/day PFOA (ammonium salt, catalog no. 77262, Sigma-Aldrich) in 0.5% Tween 20, or 50 mg/kg/day Wy-14,643 (catalog no. C7081, Sigma-Aldrich) in 0.5% methylcellulose, along with vehicle controls. All dosing solutions were freshly prepared each day. At the end of the dosing period, animals were euthanized by CO_2_ asphyxiation and tissue collected from the left lobe of the liver for preparation of total RNA. Tissue prepared for histology was collected from the same group of animals used for microarray analysis and was taken from a section adjacent to that utilized for RNA preparation.

### 2.2. RNA Preparation

Collected tissue (≤50 mg) was immediately placed in 1 mL RNA*later* (Applied Biosystems/Ambion, Austin, TX) and stored at −20°C. RNA preparations for microarray analysis were then completed by homogenizing the tissue in 1 mL TRI reagent (Sigma Chemical) followed by processing through isopropanol precipitation according to the manufacturer's instructions. The resulting pellets were washed with 80% ethanol and resuspended in RNase free water (Applied Biosystems/Ambion). Preparations were further purified by passing approximately 100 *μ*g per sample through RNeasy spin columns (Qiagen, Valencia, CA). RNA for PCR analysis was prepared using the *mir*VANA miRNA isolation kit (Applied Biosystems/Ambion) according to the manufacturer's protocol without further enrichment for small RNAs. All samples used in the study were quantified using a NanoDrop ND-1000 spectrophotometer (NanoDrop Technologies, Wilmington, DE) and quality evaluated using a 2100 Bioanalyzer (Agilent, Palo Alto, CA). Only samples with an RNA Integrity number of at least 8.0 (2100 Expert software, version B.01.03) were included in the study [35].

### 2.3. Histological Examination of Tissue

Following overnight fixation in Bouins fixative, collected tissue was washed three times in PBS, dehydrated to 70% ethanol, and stored at 4°C until use. On the day of embedding, the tissue was dehydrated through an ethanol gradient to 100% ethanol and paraffin embedded using standard techniques. Five micron sections were then prepared using a rotary microtome prior to routine staining with hematoxylin and eosin.

### 2.4. Gene Profiling

Microarray analysis was conducted at the U.S. EPA NHEERL Toxicogenomics Core Facility using Affymetrix GeneChip 430_2 mouse genome arrays according to the protocols recommended by the manufacturer (Affymetrix, Santa Clara, CA). Biotin-labeled cRNA was produced from 5 ug total RNA using Enzo Single-Round RNA Amplification and Biotin Labeling System (Cat. no. 42420-10, Enzo Life Sciences Inc, Farmingdale, NY), quantified using an ND-1000 spectrophotometer, and evaluated on a 2100 Bioanalyzer after fragmentation. To minimize technical day to day variation, labeling and hybridization for all samples were conducted as a single block. Following overnight hybridization at 45°C in an Affymetrix Model 640 GeneChip hybridization oven, the arrays were washed and stained using an Affymetrix 450 fluidics station and scanned on an Affymetrix Model 3000 scanner. Raw data (Affymetrix Cel files) were obtained using Affymetrix GeneChip Operating Software (version 1.4). This software also provided summary reports by which array QA metrics were evaluated including average background, average signal, and 3′/5′ expression ratios for spike-in controls, *β*-actin, and GAPDH. Only arrays of high quality based on low background levels as well as expected 3′/5′ expression ratios for the spike-in controls, *β*-actin, and GAPDH were included in the study. Data are available through the Gene Expression Omnibus at the National Center for Biotechnology Information (http://www.ncbi.nlm.nih.gov/geo) as accession numbers GSE22871.

### 2.5. PCR Confirmation of Results

Real-time PCR analysis of selected genes was conducted using 2 micrograms of total RNA. All samples were initially digested using 2 units DNaseI (no. M6101, Promega Corporation, Madison, WI) for 30 min at 37°C followed by 10 min at 65°C in a buffer containing 40 mM Tris (pH 8.0), 10 mM MgSO_4_, and 1 mM CaCl_2_. The RNA was then quantified using a Quant-iT RiboGreen RNA assay kit according to the manufacturer's protocol (no.R11490, Invitrogen Corporation, Carlsbad, CA) and approximately 1.5 ug RNA reverse transcribed using a High Capacity cDNA Archive Kit according to the provided protocol (no. 4322171, Applied Biosystems, Foster City, CA). Amplification was performed on an Applied Biosystems model 7900HT Fast Real-Time PCR System in duplicate using 25 ng cDNA and TaqMan Universal PCR Master Mix (no.4304437, Applied Biosystems) in a total volume of 12 *μ*L according to the protocol supplied by the manufacturer. Glyceraldehyde-3-phosphate dehydrogenase (*Gapdh*, Entrez no. 14433), which was uniformly expressed among all samples (cycle threshold deviation less than 0.35), was used as an endogenous reference gene. The following TaqMan assays (Applied Biosystems) were included in the study: *Gapdh* (no. Mm99999915_g1), *Srebf2* (no. Mm01306293_m1), *P* *p* *a* *r* *g* *c*1*a* (Mm0047183_m1), *Nfe2l2 (Mm00477784_m1), Ndufa5* (Mm00471676), *Lss* (no. Mm00461312_m1), *Cyp4a14* (no. Mm00484132_m1), *Cyp7a1* (no. Mm00484152_m1), and *Cyp2b10* (no. Mm00456591_m1). Fold change was calculated using the 2^-ΔΔC^T method of Livak and Schmittgen [36].

### 2.6. Data Analysis

Body and liver weight data were analyzed by strain using a one-way ANOVA. Individual treatment contrasts were assessed using a Tukey Kramer HSD test (*P* ≤ .05) (JMP 7.0 (SAS, Cary, NC). Microarray data were summarized, background adjusted, and quantile normalized using Robust Multichip Average methodology (RMA Express, ver. 1.0). Prior to statistical analysis, microarray data were filtered to remove probe sets with weak or no signal. Data were analyzed for each strain using a one-way ANOVA across dose (Proc GLM, SAS ver. 9.1, Cary, NC). Individual treatment contrasts were evaluated using a pairwise *t*-test of the least square means. Significant probe sets (*P* ≤ .0025) were evaluated for relevance to biological pathway and function using Ingenuity Pathway Analysis software (http://analysis.ingenuity.com/) and DAVID functional annotation software [37]. Duplicate probe sets were resolved using minimum *P*-value. Data were further evaluated without statistical filtering using Gene Set Enrichment Analysis (GSEA) software available from the Broad Institute [38]. Hierarchical clustering and heat maps were generated using Eisen Lab Cluster and Treeview software (version 2.11).

## 3. Results

### 3.1. Necropsy and Histopathology

**Figure 1:**
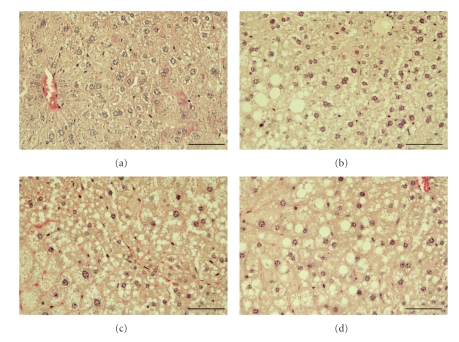
Hematoxylin-and eosin-stained tissue sections from control and PFOS treated mice. Control WT and Null mice are shown in panels (a) and (b), respectively. WT and null mice treated with 10 mg/kg/day PFOS are shown in panels (c) and (d), respectively. Vacuole formation was observed in sections from treated WT mice, and in sections from control and treated Null mice. Mice exposed to 3 mg/kg/day PFOS were similar to controls (data not shown). Bar = 50 *μ*m.

Liver weight increased at the highest dose of PFOS in both WT and Null animals (Table 1). Histological changes were also noted. Vacuole formation was observed in tissue sections from treated WT mice, as well as in sections from control and treated Null mice (Figure 1). The origin of these vacuoles was not fully apparent. Kudo and Kawashima [28] reported that chronic exposure to PFOA can induce fatty liver in mice due to altered triglyceride transport; hence, vacuolization in the current study may be the result of similar changes in WT mice. In Null mice, vacuole formation may also reflect increased triglyceride retention due to reduced hepatic fatty acid catabolism. Furthermore, our group has suggested that a certain degree of vacuolization may be unrelated to triglyceride retention in PFOA-exposed Null mice [29]. It is possible therefore, that hepatic vacuolization might be associated with the liver weight increase observed in treated Null animals.

### 3.2. Gene Profiling

**Figure 2:**
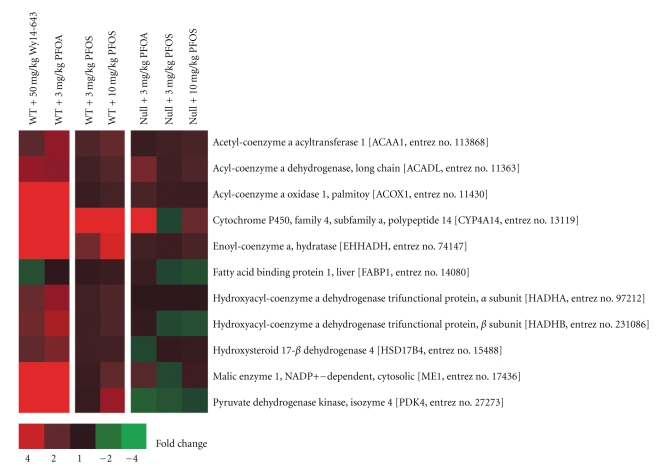
Expression of a group of well characterized markers of PPAR*α* transactivation in WT and Null mice. The response to PFOS in WT mice was less robust than that previously observed for either PFOA or Wy14,643. Red or green correspond to average up- or down- regulation, respectively.

Based on the number of genes significantly altered by PFOS (*P* ≤ .0025), gene expression changes in WT mice were more evident at the higher dose of PFOS compared to the lower dose. This was in contrast to changes observed in Null mice where the number of transcripts influenced by PFOS was similar across either dose group. Hence, certain PPAR*α*-independent effects were found to be robust in Null mice even at the lowest dose of PFOS. This pattern of gene expression also varied from that previously observed by our group for PFOA where only moderate changes were found in Null mice compared to WT animals [1] (Table 2). By examining the expression of a small group of well characterized markers of PPAR*α* transactivation, PFOS also appeared to be a less robust activator of murine PPAR*α* than PFOA (Figure 2), a conclusion formerly reported by others [18, 39, 40]. 

**Figure 3:**
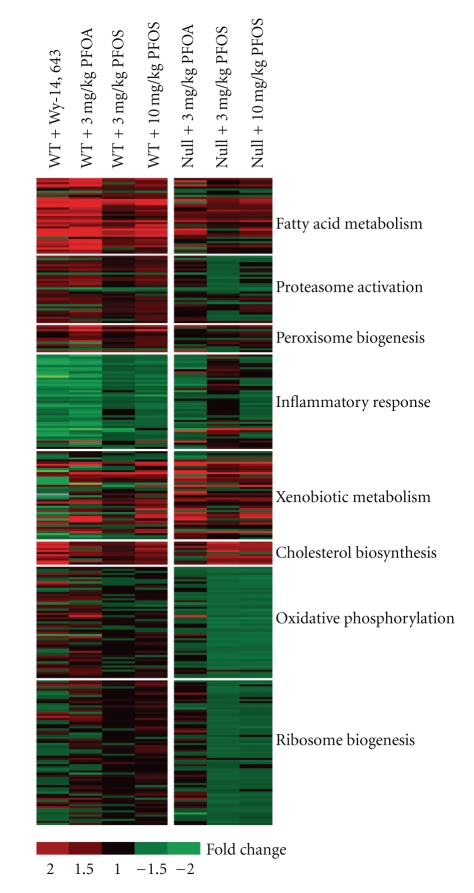
Functional categories of genes modified by PFOS in WT and Null mice. In WT mice, PFOS altered the expression of genes related to a variety of PPAR*α*-regulated functions including lipid metabolism, peroxisome biogenesis, proteasome activation, and the inflammatory response. Genes affected in both WT and Null mice consisted of transcripts related to lipid metabolism, inflammation, and xenobiotic metabolism. Several categories of genes were uniquely regulated by PFOS in Null mice including up-regulation of genes in the cholesterol biosynthesis pathway as well as modest down-regulation of genes associated with oxidative phosphorylation and ribosome biogenesis. Red or green corresponds to average up- or down- regulation, respectively.

In WT mice, PFOS modified the expression of genes related to a variety of PPAR*α*-regulated functions including lipid metabolism, peroxisome biogenesis, proteasome activation, and the inflammatory response. Genes affected in both WT and Null mice consisted of transcripts related to lipid metabolism, inflammation, and xenobiotic metabolism, including the CAR inducible gene, *Cyp2b10*. It should be stressed, however, that those changes associated with the inflammatory response in Null mice were modest and were only apparent within the context of similar but more robust changes in WT mice. Several categories of genes were also uniquely regulated in Null mice by PFOS including up-regulation of genes in the cholesterol biosynthesis pathway, along with modest down-regulation of genes (<1.5 fold change) associated with oxidative phosphorylation and ribosome biogenesis (Figure 3). Changes related to ribosome biogenesis were particularly subtle and were identified by the computational method provided by GSEA using the complete set of expressed genes without statistical filtering. This approach allowed for an a priori set of genes to be evaluated for significant enrichment without regard for the statistical significance of individual genes. Among the changes uniquely induced by PFOS in Null mice was up-regulation of *Cyp7a1*, an important gene related to bile acid/cholesterol homeostasis. Data for individual genes are provided in Tables 3–10.

### 3.3. PCR Confirmation

**Figure 4:**
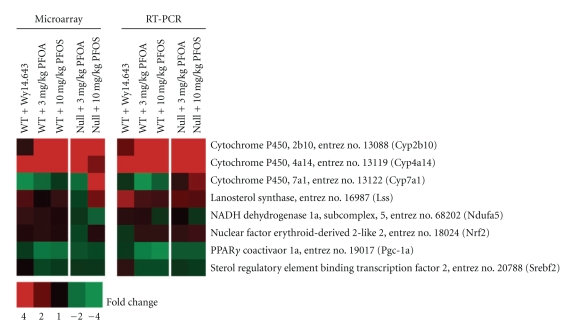
Microarray and Real-time PCR analysis of selected genes. Data from both assays were in close agreement. Small changes in *Ndufa5 *expression, a gene which encodes for a subunit of mitochondrial respiratory chain complex I, could not be confirmed by RT-PCR. As predicted based on microarray analysis, PFOS did not appear to up-regulate the expression of *Srebf2*, *P* *p* *a* *r* *g* *c*1*a* *(Pgc-1a)*, or *Nfe2l2* (*Nrf2*) in WT or Null mice. Red or green correspond to average up- or down- regulation, respectively.

The results from real-time RT-PCR analysis of selected genes are summarized, along with the corresponding results from the microarray analysis, in Figure 4. The data from both assays were in close agreement. It should be pointed out that while up-regulation of *Cyp2b10* was confirmed in treated WT and Null mice, it remained a low copy number transcript in these animals. Down-regulation of *Ndufa5*, a gene which encodes for a subunit of mitochondrial respiratory chain complex I, could not be confirmed in treated Null mice. This result, however, was not surprising because the changes associated with oxidative phosphorylation in the current study were small and, therefore, difficult to detect given the technical variation normally associated with real-time PCR. As predicted based on the microarray results, PFOS did not appear to up-regulate the expression of *Srebf2*, *P* *p* *a* *r* *g* *c*1*a*, or *Nfe2l2* (*Nrf2*) in either WT or Null mice.

## 4. Discussion

In the current study, exposure to PFOS induced both PPAR*α*-dependent and PPAR*α*-independent effects in the murine liver. In WT mice, the observed changes were primarily indicative of a weak PPAR*α* activator. As such, PFOS induced hepatomegaly and altered the expression of genes related to a number of biological functions known to be regulated by PPAR*α* including lipid metabolism, peroxisome biogenesis, proteasome activation, and the inflammatory response [41–45]. These data are also in agreement with previous studies done in either the adult or fetal rodent [46–50]. Among those effects found to be independent of PPAR*α* was altered expression of genes associated with xenobiotic metabolism, including up-regulation of the CAR inducible gene, *Cyp2b10*. Such changes, which were found in both WT and Null mice, were also consistent with results previously reported by our group for PFOA [32, 33]. Although xenobiotic metabolism can be regulated by more than one nuclear receptor [51], the ability of PFOA or perfluorodecanoic acid (PFDA) to activate CAR has been demonstrated in experiments using multiple receptor-null mouse models [31]; therefore, it is likely that PFOS functions as an activator of CAR as well. Additional PPAR*α*-unrelated effects were further indicated by regulation of a group of genes associated with lipid metabolism and inflammation in both WT and Null mice. As suggested for mice exposed to PFOA [1, 33], such changes could be due to activation of either PPAR*γ* and/or PPAR*β*/*δ*. Indeed, studies done using transient transfection reporter cell assays indicate that PFOS and PFOA have the potential to modestly activate other PPAR isotypes. [39, 40]. Furthermore, peroxisome proliferation, a hallmark of PPAR*α* transactivation, can also be induced in the rodent liver by activating PPAR*γ* and/or PPAR*β*/*δ* [52]; hence, a degree of functional overlap might be expected among the PPAR isotypes. Particularly noteworthy were PPAR*α*-independent effects that were unique to Null mice since they were not previously observed in mice treated with PFOA [1, 33]. These included modified expression of genes associated with ribosome biogenesis, oxidative phosphorylation, and cholesterol biosynthesis. While activation of PPAR*α* has been linked to changes in cholesterol homeostasis [19] and oxidative phosphorylation [53], it should be stressed that such changes were not simply the result of targeted disruption of PPAR*α* because they were observed in treated animals over and above those effects which occurred in Null controls. Moreover, in the current study, genes linked to cholesterol biosynthesis were found to be up-regulated in Null mice, an effect that mirrored changes previously reported in WT mice treated with the PPAR*α* agonist, Wy 14,643 [1]. 

Recognition that PPAR ligands can induce “off-target” effects is not new (for review, see [54]). It is not clear, however, whether the effects described for Null mice in the current study were the result of modified activity of transcription regulators, which only became apparent in the absence of PPAR*α* signaling, or whether these changes represent some other aspect of murine metabolism affected by PFOS. Of interest was up-regulation of *Cyp7a1*. This gene encodes for an enzyme responsible for the rate limiting step in the classical pathway of hepatic bile acid biosynthesis and is important for bile acid/cholesterol homeostasis [55]. While targeted disruption of PPAR*α* does not appear to alter basal levels of *Cyp7a1* [56], PPAR*α* agonists such as, fibrates can reduce both *Cyp7a1* gene expression and bile acid biosynthesis in wild-type rodents [57] possibly by interfering with promoter binding of HNF4 [58]. Regulation of *Cyp7a1* is often associated with the liver X receptor (LXR) [59] but it is tightly controlled by multiple pathways and may be positively regulated by the pregnane X receptor (PXR) [60] and the retinoid X receptor (RXR) as well [61]. While the two LXR subtypes, LXR*α* and LXR*β*, are lipogenic and play a key role in regulating cholesterol homeostasis [62, 63], they are not thought to be positive regulators of genes in the cholesterol biosynthesis pathway [64]. 

Additional signaling pathways that may contribute to the effects observed in Null mice include pathways regulated by Srebf2 (Srebp2) and PPARGC1*α* (PGC-1*α*). Srebf2 is one member of a group of membrane-bound transcription factors that play an important role in maintaining lipid homeostasis. SREBF2 is best known for positively regulating cholesterol synthesis in the liver and other tissues (Horton et al., 1998). While decreased nuclear abundance of SREBP2 has been linked to increased hepatic PPAR*α* activity in rats [65], a PPAR*α*-independent mechanism of action has been suggested in mice as well which, in combination with increased expression of CYP7a1, may paradoxically also function via decreased SREBF2 signaling [66]. It should be noted that transcript levels of *Srebf2* were not affected in the current study nor was PFOS found to alter *Srebf2* expression in cultured chicken hepatocytes [67], although such changes are not necessarily required for transcription factor regulation. Rather than functioning as a transcription factor like SREBP2, PPARGC-1*α* is a transcription coactivator that was first described as a moderator of PPAR*γ*-induced adaptive thermogenesis in brown adipose tissue [68]. PPARGC-1*α* is now known to regulate various aspects of energy metabolism in different tissues by interacting with a host of transcription factors, including PPAR*α* [69, 70]. Certain PPAR ligands have been shown to inhibit oxidative phosphorylation [71–74] and Walters et al. [75] recently reported that high doses of PFOA could modify mitochondrial function in rats via a pathway involving PPARGC-1*α*. Unlike their results, however, PFOS did not induce a change in expression of *Ppargc*-1*α* or its downstream target, *Nrf2*, in the current study. Cellular regulation of metabolism, however, is complex and there are a number of potentially interrelated signaling pathways, including HNF4*α* [76] and TOR [77], that based on their biological function could theoretically be linked to the effects observed in PFOS-treated Null mice. Given the diversity of effects observed in the current study, it is likely that more than one signaling pathway is responsible for the biological activity reported for PFOS. 

Because certain effects were found only in Null mice, their relevance to the toxicity of PFOS is not clear. Although the developmental toxicity of PFOS has been shown to be independent of PPAR*α* in murine neonates [34], it has also been suggested that rather than causing primary alterations to the murine transcriptome, PFOS may alter the physicochemical properties of fetal lung surfactant as the critical event related to toxicity in these animals [78–80]. It should also be stressed that in Null animals the magnitude of change found for certain effects was small, hence, the reported effects in the current study were subtle. On the other hand, these data serve to reinforce two recurring themes regarding the biological activity of PFAAs. First, as a class of compounds, the activity of PFAAs may be quite variable. Differences exist among PFAAs with regard to chain length and functional group which influence, not only the elimination half-life of assorted PFAAs [4, 7] and their ability to activate PPAR*α* [18], but potentially their ability to modify the function of other transcription regulators as well. Second, the biological activity of PFAAs is likely to differ from that observed for fibrate pharmaceuticals, the most commonly studied ligands of PPAR*α*. While much has been learned from studies using fibrate-exposed PPAR*α*-null and PPAR*α*-humanized mice regarding the relevance of chronic PPAR*α* activation to liver tumor formation in humans [22], additional information concerning the biological activity of specific PFAAs remains relevant for risk assessment. 

In summary, PFOS is a PPAR*α* agonist that is capable of inducing a variety of PPAR*α*-independent effects in WT and Null mice, although the toxicological relevance of these changes is uncertain. A number of these effects such as, altered expression of genes involved in lipid metabolism, inflammation, and xenobiotic metabolism were observed in both WT and Null animals, and were consistent with prior studies done with either PFOS or PFOA. Other effects involving genes associated with ribosome biogenesis, oxidative phosphorylation, and cholesterol biosynthesis were unique to Null mice and may represent targeted signaling pathways not yet described for certain PFAAs.

## Figures and Tables

**Table 1:** Average body weight and liver weight of control and PFOS-treated mice on the day of tissue collection.^1^

Dose group	WT			Null		
	Body weight	Total liver weight	Relative liver weight	Body weight	Total liver weight	Relative liver weight
0 mg/kg	28.3 ± .0	1.21 ± 0.17	0.043 ± 0.014	30.3 ± 1.3	1.04 ± 0.06	0.034 ± 0.003
3 mg/kg	26.2 ± 1.5	1.12 ± 0.18	0.043 ± 0.002	28.0 ± 1.2	1.20 ± 0.05	0.043 ± 0.001
10 mg/kg	31.4 ± 1.5	1.98 ± 0.11*	0.062 ± 0.003*	30.2 ± 1.7	1.48 ± 0.16*	0.049 ± 0.012*

^1^Data are mean ± SE, *Significantly different than control (*P* ≤ .05).

**Table 2:** Number of fully annotated genes altered by PFOS, PFOA^1^, or Wy-14,643^1^ in wild-type and PPAR*α*-null mice (*P* ≤ .0025)^2^.

	POS		PFOA	Wy 14,643
	3 mg/kg/day	10 mg/kg/day	3 mg/kg	50 mg/kg/day
Wild-type	81	906	879	902
PPAR*α*-null	630	808	176	10

^1^From Rosen et al. (2008), ^2^ Based on Ingenuity Pathways Analysis database.

**Table 3:** Average fold change for genes related to lipid metabolism in wild-type and PPAR*α*-null male mice following a seven-day exposure to Wy-14,643^1^, PFOA^1^, or PFOS.

				WT				Null	
Symbol	Gene name	Entrez no.	Wy14,643 50 mg/kg	PFOA 3 mg/kg	PFOS 3 mg/kg	PFOS 10 mg/kg	PFOA 3 mg/kg	PFOS 3 mg/kg	PFOS 10 mg/kg
ACAA1	acetyl-CoA acyltransferase 1	113868	1.89	2.92	1.61	2.10**	1.22	1.37	1.53*
ACAA1B	acetyl-CoA acyltransferase 1B	235674	2.38	2.70	1.49	1.40**	3.00	1.09	1.19*
ACAD10	acyl-CoA dehydrogenase, member 10	71985	1.51	2.39	−1.18	1.38**	−1.01	1.05	1.20*
ACADL	acyl-CoA dehydrogenase, long chain	11363	3.03	2.86	1.40	1.68**	2.50	1.34	1.59**
ACADM	acyl-CoA dehydrogenase, C-4 to C-12	11364	1.70	1.30	1.21	1.31**	1.06	1.11	1.10
ACADS	acyl-CoA dehydrogenase, C-2 to C-3	66885	1.03	1.52	1.22	1.31*	−1.13	−1.12	−1.08
ACADSB	acyl-CoA dehydrogenase, short/branched	66885	−1.56	−1.64	−1.04	−1.39**	−1.26	1.00	−1.23
ACADVL	acyl-CoA dehydrogenase, very long chain	11370	1.92	1.80	1.44	1.49**	1.16	1.04	1.12
ACAT1	acetyl-CoA acetyltransferase 1	101446	−1.01	1.10	1.45	1.36*	−1.55	−1.05	−1.17
ACAT2	acetyl-CoA acetyltransferase 2	110460	2.59	1.68	1.14	1.34*	1.26	1.58	1.69**
ACOT1	acyl-CoA thioesterase 1	26897	19.48	73.06	3.27	6.82**	2.95	1.53	2.02
ACOT3	acyl-CoA thioesterase 3	171281	2.55	32.83	2.42	6.41**	−1.59	1.46	1.86
ACOT2	acyl-CoA thioesterase 2	171210	3.83	19.29	1.91	7.32**	1.78	1.25	1.52
ACOX1	acyl-CoA oxidase 1	11430	5.65	7.17	1.23	1.49**	1.51	1.30	1.29**
ACSL1	acyl-CoA synthetase long- chain member1	14081	1.34	2.36	1.28	1.36**	1.01	1.31	1.30
ACSL3	acyl-CoA synthetase long- chain member3	74205	2.25	1.90	1.28	1.69**	1.11	1.77	1.63
ACSL4	acyl-CoA synthetase long- chain member4	50790	1.95	2.00	1.03	1.42*	1.51	1.34	1.29
ACSL5	acyl-CoA synthetase long- chain member5	433256	3.06	2.76	1.24	1.31**	1.38	1.23	1.28
ALDH1A1	aldehyde dehydrogenase 1, member A1	11668	1.56	1.59	1.07	1.12**	1.22	1.16	1.17
ALDH1A7	aldehyde dehydrogenase 1, A7	26358	1.83	1.86	1.12	1.24*	1.55	1.26	1.35
ALDH3A2	aldehyde dehydrogenase 3, member A2	11671	3.65	7.72	2.10	3.80**	2.30	1.73	2.20**
ALDH9A1	aldehyde dehydrogenase 9, member A1	56752	1.80	1.91	1.27	1.50**	1.21	1.05	1.11*
CPT1B	carnitine palmitoyltransferase 1B (muscle)	12896	2.29	1.50	1.23	2.69**	−1.00	1.13	1.11
CPT2	carnitine palmitoyltransferase II	12896	1.33	2.54	1.58	2.03**	1.44	1.15	1.34
CYP4A14	cytochrome P450, 4, a, polypeptide 14	13119	75.38	103.48	11.26	12.28**	12.75	−1.09	2.22
DCI	dodecenoyl-CoA delta isomerase	13177	2.91	4.55	1.90	2.38**	1.99	1.04	1.38*
ECH1	enoyl CoA hydratase 1, peroxisomal	51798	3.27	5.23	1.93	2.49**	2.10	1.16	1.39
EHHADH	enoyl-CoA, hydratase	74147	27.89	22.11	2.37	4.34**	1.37	1.32	1.52*
FABP1	fatty acid binding protein 1, liver	14080	−1.27	1.02	1.11	1.24**	1.25	−1.09	−1.23
HADHA	Trifunctional protein, alpha unit	97212	2.13	2.95	1.37	1.65**	1.01	1.06	1.02
HADHB	Trifunctional protein, beta unit	231086	2.33	3.43	1.37	1.60**	1.08	−1.15	−1.28*
HSD17B4	hydroxysteroid (17-beta) dehydrogenase4	15488	2.03	2.56	1.34	1.45**	−1.13	1.12	1.20*
SLC27A1	solute carrier 27, member 1	26457	9.14	8.22	−1.02	1.14*	−1.57	1.04	1.04
SLC27A2	solute carrier 27, member 2	26458	1.48	1.80	1.19	1.16**	1.33	1.10	1.05
SLC27A4	solute carrier 27, member 4	26569	1.87	1.91	1.04	1.31**	−1.03	1.09	1.07

^1^From Rosen et al. (2008),

*Significantly different than control (*P* ≤ .03),

**Significantly different than control (*P* ≤ .0025)

**Table 4:** Average fold change for genes related to proteasome biogenesis in wild-type and PPAR*α*-null male mice following a seven-day exposure to Wy-14,643^1^, PFOA^1^, or PFOS.

					WT			Null	
Symbol	Gene name	Entrez no.	Wy14,643 50 mg/kg	PFOA 3 mg/kg	PFOS 3 mg/kg	PFOS 10 mg/kg	PFOA 3 mg/kg	PFOS 3 mg/kg	PFOS 10 mg/kg
PSMA1	proteasome unit, alpha type, 1	26440	1.61	1.38	1.15	1.31*	1.17	−1.29	−1.34
PSMA2	proteasome unit, alpha type, 2	19166	−1.46	−1.15	1.09	1.23**	−1.34	−1.20	−1.07
PSMA3	proteasome unit, alpha type, 3	19167	1.33	1.22	1.12	1.14	1.28	−1.13	−1.17
PSMA4	proteasome unit, alpha type, 4	26441	1.19	1.32	1.10	1.19*	1.01	−1.04	1.05
PSMA5	proteasome unit, alpha type, 5	26442	1.67	1.59	1.12	1.26**	1.15	−1.12	1.09
PSMA6	proteasome unit, alpha type, 6	26443	1.20	1.29	1.14	1.24**	1.06	−1.14	−1.06
PSMA7	proteasome unit, alpha type, 7	26444	1.47	1.60	1.23	1.53**	1.23	−1.12	1.11
PSMB1	proteasome unit, beta type, 1	19170	1.09	1.29	1.07	1.28*	1.04	−1.17	1.13*
PSMB10	proteasome unit, beta type, 10	19171	−1.42	−1.48	−1.25	−1.19	−1.57	−1.14	−1.21**
PSMB2	proteasome unit, beta type, 2	26445	1.33	1.48	1.05	1.31**	1.02	−1.20	1.05
PSMB3	proteasome unit, beta type, 3	26446	1.22	1.47	1.21	1.36**	1.04	−1.37	−1.20
PSMB4	proteasome unit, beta type, 4	19172	1.59	1.65	1.27	1.55**	1.22	−1.12	1.09
PSMB5	proteasome unit, beta type, 5	19173	1.34	1.74	1.04	1.24**	1.02	−1.15	1.03
PSMB6	proteasome unit, beta type, 6	19175	1.54	1.83	1.08	1.24*	1.19	−1.23	−1.09
PSMB7	proteasome unit, beta type, 7	19177	1.46	1.33	1.07	1.15**	1.13	−1.17	−1.09
PSMB8	proteasome unit, beta type, 8	16913	−1.61	−2.00	−1.44	−1.51	−1.38	−1.23	−1.45**
PSMB9	proteasome unit, beta type, 9	16912	1.24	−1.12	−1.31	−1.09	−1.10	−1.11	−1.30**
PSMC1	proteasome 26S unit, ATPase, 1	19179	1.44	1.00	1.19	1.15*	1.11	−1.06	1.01
PSMC6	proteasome 26S unit, ATPase, 6	67089	1.18	1.21	1.09	−1.02	1.07	1.14	−1.16
PSMD1	proteasome 26S unit, non-ATPase, 1	70247	1.20	1.22	1.15	1.25**	1.09	1.03	1.15
PSMD11	proteasome 26S unit, non-ATPase, 11	69077	1.56	1.38	1.09	1.26*	−1.17	1.16	1.32
PSMD12	proteasome 26S unit, non-ATPase, 12	66997	1.34	1.27	1.10	1.14	1.20	−1.03	1.04
PSMD13	proteasome 26S unit, non-ATPase, 13	23997	1.21	1.38	1.14	1.26*	−1.03	−1.38	−1.42**
PSMD14	proteasome 26S unit, non-ATPase, 14	59029	−1.39	−1.42	1.17	1.31*	1.31	1.01	1.17
PSMD2	proteasome 26S unit, non-ATPase, 2	21762	1.34	1.32	1.14	1.24*	1.10	1.09	1.30**
PSMD3	proteasome 26S unit, non-ATPase, 3	22123	−1.35	−1.19	1.17	1.29*	1.08	1.04	1.22*
PSMD4	proteasome 26S unit, non-ATPase, 4	19185	1.31	1.92	1.19	1.38**	1.03	−1.07	1.17*
PSMD6	proteasome 26S unit, non-ATPase, 6	66413	1.17	1.33	1.10	1.14*	1.07	−1.06	1.04
PSMD7	proteasome 26S unit, non-ATPase, 7	17463	1.13	1.27	1.13	1.24*	1.02	−1.19	−1.22*
PSMD8	proteasome 26S unit, non-ATPase, 8	57296	1.68	1.24	1.03	1.30**	1.16	−1.15	−1.00
PSME1	proteasome activator unit 1	19186	1.22	−1.00	−1.05	1.32**	1.27	−1.10	−1.09
VCP	valosin−containing protein	269523	1.40	1.49	1.04	1.12	1.07	1.13	1.21**

^1^From Rosen et al. (2008),

*Significantly different than control (*P* ≤ .03),

**Significantly different than control (*P* ≤ .0025).

**Table 5:** Average fold change for genes related to peroxisome biogenesis in wild-type and PPAR*α*-null male mice following a seven-day exposure to Wy-14,643^1^, PFOA^1^, or PFOS.

					WT			Null	
Symbol	Gene name	Entrez no.	Wy14,643 50 mg/kg	PFOA 3 mg/kg	PFOS 3 mg/kg	PFOS 10 mg/kg	PFOA 3 mg/kg	PFOS 3 mg/kg	PFOS 10 mg/kg
PECI	peroxisome D3, D2-enoyl- CoA isomerase	23986	1.73	3.15	1.61	1.87**	1.96	1.42	1.57**
PEX1	peroxisomal biogenesis factor 1	71382	1.25	1.84	1.07	1.21**	−1.02	1.10	1.14*
PEX11A	peroxisomal biogenesis factor 11 alpha	18631	1.80	6.71	1.70	2.99**	1.04	−1.09	−1.11
PEX12	peroxisomal biogenesis factor 12	103737	1.07	1.36	1.11	1.17*	1.09	1.17	1.30*
PEX13	peroxisomal biogenesis factor 13	72129	1.04	1.58	1.01	1.09	1.02	1.09	1.16*
PEX14	peroxisomal biogenesis factor 14	56273	1.06	1.24	1.03	1.25*	1.03	1.05	1.13
PEX16	peroxisomal biogenesis factor 16	18633	1.51	1.44	1.13	1.33**	−1.00	−1.12	−1.03
PEX19	peroxisomal biogenesis factor 19	19298	1.61	2.25	1.19	1.36**	1.12	1.15	1.32**
PEX26	peroxisomal biogenesis factor 26	74043	−1.32	−1.86	1.01	1.26	1.01	1.29	1.10
PEX3	peroxisomal biogenesis factor 3	56535	1.50	1.77	1.13	1.37**	−1.05	1.09	1.20*
PEX6	peroxisomal biogenesis factor 6	224824	1.08	−1.06	1.12	1.16	1.30	−1.08	1.09
PXMP2	peroxisomal membrane protein 2	19301	−1.22	−1.29	−1.08	−1.20*	−1.28	−1.13	−1.06
PXMP4	peroxisomal membrane protein 4	59038	1.62	2.09	1.61	1.62*	1.99	−1.03	1.01

^1^From Rosen et al. [1],

*Significantly different than control (*P* ≤ .03),

**Significantly different than control (*P* ≤ .0025).

**Table 6:** Average fold change for genes related to the inflammatory response in wild-type and PPAR*α*-null male mice following a seven-day exposure to Wy-14,643^1^, PFOA^1^, or PFOS.

					WT			Null	
Symbol	Gene name	Entrez no.	Wy14,643 50 mg/kg	PFOA 3 mg/kg	PFOS 3 mg/kg	PFOS 10 mg/kg	PFOA 3 mg/kg	PFOS 3 mg/kg	PFOS 10 mg/kg
APCS	amyloid P component, serum	20219	−1.50	−2.33	−1.23	−1.28	−1.19	1.41	1.13
C1QA	complement component 1QA	12259	−1.75	−1.40	−1.13	−1.17	−1.31	−1.24	−1.34**
C1R	complement component 1r	50909	−2.67	−1.78	−1.15	−1.23*	−1.22	1.16	−1.17*
C1S	complement component 1s	317677	−3.73	−2.53	−1.14	−1.62**	−1.52	1.06	−1.11
C2	complement component 2	12263	−2.56	−1.91	−1.37	−1.32*	−1.18	1.10	1.11
C3	complement component 3	12266	−1.41	−1.41	−1.04	−1.04	−1.22	1.13	1.08*
C4B	complement component 4B	12268	−2.35	−2.15	−1.08	−1.28	−1.91	1.15	−1.13
C4BP	complement component 4 binding prot	12269	−1.86	−1.82	−1.11	−1.19	1.02	1.39	1.13
C6	complement component 6	12274	−2.66	−1.27	−1.35	−1.08	1.90	1.12	1.06
C8A	complement component 8, alpha	230558	−3.62	−1.94	−1.17	−1.31*	−1.17	1.19	1.04
C8B	complement component 8, beta	110382	−5.25	−2.99	−1.20	−1.60**	−1.12	1.11	1.02
C8G	complement component 8, gamma	69379	−1.59	−1.35	−1.05	−1.17*	−1.34	−1.10	−1.17**
C9	complement component 9	12279	−2.12	−2.64	−1.35	−1.58**	−1.46	1.08	−1.19*
CFB	complement factor B	14962	−1.81	−1.77	−1.07	−1.26	−1.39	1.07	−1.11
CFH	complement factor H	12628	−2.39	−2.30	−1.19	−1.62	−1.76	1.45	−1.35
CFI	complement factor I	12630	−1.63	−1.77	−1.06	−1.15	−1.06	1.12	1.04
CRP	C−reactive protein	12944	−1.33	−1.39	−1.01	−1.15*	1.32	1.14	1.13
CTSC	cathepsin C	13032	−1.56	−2.52	1.01	−1.36	−1.96	1.04	−1.35
F10	coagulation factor X	14058	−1.62	−1.42	−1.09	−1.13	−1.00	1.07	−1.07
F11	coagulation factor XI	109821	−2.17	−2.68	−1.41	−2.08**	−1.08	−1.08	−1.34*
F12	coagulation factor XII	58992	−1.22	−1.35	−1.05	−1.14	−1.21	−1.07	−1.12*
F13B	coagulation factor XIII, B polypeptide	14060	−1.41	−1.54	−1.11	−1.22**	1.02	1.02	−1.12
F2	coagulation factor II (thrombin)	14061	−1.19	−1.20	−1.02	−1.13*	−1.10	1.02	−1.02
F5	coagulation factor V	14067	−1.78	−1.53	−1.09	−1.44*	−1.41	1.08	−1.34*
F7	coagulation factor VII	14068	−2.68	−2.15	−1.09	−1.46**	−1.23	1.03	−1.03
F9	coagulation factor IX	14071	−1.42	−1.43	−1.02	−1.39*	−1.33	1.07	−1.19
FGA	fibrinogen alpha chain	14161	−1.27	−1.75	1.00	−1.12	−1.07	1.05	−1.07
FGB	fibrinogen beta chain	110135	−1.32	−1.97	1.03	−1.15	−1.25	1.08	−1.07
FGG	fibrinogen gamma chain	99571	−1.14	−1.68	1.02	−1.15*	−1.08	1.04	−1.06
KLKB1	kallikrein B, plasma (Fletcher factor) 1	16621	−1.58	−1.76	−1.09	−1.39*	−1.05	−1.03	−1.18*
LUM	lumican	17022	−1.34	−1.27	1.02	−1.20*	−1.66	1.03	−1.27
MASP1	Mannan- binding lectin1	17174	−1.23	−1.62	−1.19	−1.18*	1.11	1.18	1.17*
MBL2	Mannose-binding lectin 2	17195	−1.77	−2.18	−1.12	−1.23*	−1.36	−1.20	−1.28**
ORM2	orosomucoid 2	18405	−1.96	−2.04	−1.26	−1.21	−1.16	1.30	1.05
PROC	protein C	19123	−1.49	−1.50	−1.02	−1.13*	−1.09	−1.01	−1.09*
SAA1	serum amyloid A1	20209	−3.71	−3.98	−2.75	1.04	−2.76	6.51	2.55
SAA2	serum amyloid A2	20210	−1.75	−1.30	−1.79	−1.29	3.05	1.44	1.22
SAA4	serum amyloid A4, constitutive	20211	−2.19	−1.45	−1.06	−1.27	−1.02	1.47	−1.05
SERPINA1	serpin peptidase inhibitor, clade A1	20701	−3.43	−2.07	−1.03	−1.05**	−1.16	1.11	−1.33
SERPINC1	serpin peptidase inhibitor, clade C1	11905	−1.19	−1.21	−1.03	−1.08*	−1.02	−1.04	−1.06*
SERPIND1	serpin peptidase inhibitor, clade D1	15160	−1.62	−1.70	−1.08	−1.25**	−1.05	1.09	1.05
SERPINE1	serpin peptidase inhibitor, clade E1	18787	1.44	9.75	1.03	1.85**	2.95	1.03	1.26*
SERPINF2	serpin peptidase inhibitor, clade F2	18816	−1.15	−1.87	1.01	−1.13*	1.02	1.12	1.05
SERPING1	serpin peptidase inhibitor, clade G1	12258	−1.23	−1.37	−1.12	−1.13	−1.07	1.12	1.02
VWF	von Willebrand factor	22371	1.06	1.12	−1.25	1.07	−1.51	1.22	1.14

^1^From Rosen et al. [1],

*Significantly different than control (*P* ≤ .03),

**Significantly different than control (*P* ≤ .0025).

**Table 7:** Average fold change for genes related to xenobiotic metabolism in wild-type and PPAR*α*-null male mice following a seven-day exposure to Wy-14,643^1^, PFOA^1^, or PFOS.

					WT			Null	
Symbol	Gene name	Entrez no.	Wy14,643 50 mg/kg	PFOA 3 mg/kg	PFOS 3 mg/kg	PFOS 10 mg/kg	PFOA 3 mg/kg	PFOS 3 mg/kg	PFOS 10 mg/kg
ADH1C	alcohol dehydrogenase 1C	11522	1.27	1.02	−1.00	1.02	−1.09	−1.02	−1.04
ADH5	alcohol dehydrogenase 5	11532	−1.18	1.10	1.09	−1.04	−1.02	1.11	1.14
ADH7	alcohol dehydrogenase 7	11529	−1.51	1.06	−1.01	−1.06	−1.71	−1.01	−1.01
ALDH1L1	aldehyde dehydrogenase 1L1	107747	−1.29	−1.85	−1.08	−1.18*	−1.41	1.76	1.68**
ALDH3B1	aldehyde dehydrogenase 3B1	67689	1.12	1.04	−1.11	1.04	1.48	−1.03	−1.11
CES1	carboxylesterase 1	12623	1.43	2.29	1.61	2.62**	3.15	4.80	4.84**
CES2	carboxylesterase 2	234671	3.37	5.75	1.03	2.29	4.25	1.41	1.74*
CYP1A1	cytochrome P450,1A1	13076	1.25	−1.93	−1.05	1.08	−1.02	1.34	1.49**
CYP1A2	cytochrome P450,1A2	13077	−1.67	−1.24	−1.13	1.10	1.26	1.15	1.25*
CYP2A4	cytochrome P450,2A4	13087	−4.26	1.33	1.08	2.01	5.82	1.28	1.57**
CYP2B10	cytochrome P450,2B10	13088	1.31	4.39	3.50	5.92*	24.20	11.34	21.66**
CYP2C55	cytochrome P450,2C55	72082	1.58	21.72	1.54	8.37*	110.35	10.57	25.18**
CYP2C37	cytochrome P450,2C37	13096	−2.42	1.57	1.39	1.48	4.09	1.53	1.68
CYP2C38	cytochrome P450, 2C38	13097	1.62	1.12	1.78	2.30**	−1.42	−1.26	1.03
CYP2C39	cytochrome P450, 2C39	13098	2.45	1.51	1.65	1.51	−1.42	1.11	−1.01
CYP2C50	cytochrome P450,2C50	107141	−2.63	1.31	1.11	1.19	1.71	1.34	1.26
CYP2C54	cytochrome P450,2C54	404195	−2.98	1.44	1.16	1.14	1.87	1.29	1.35**
CYP2C70	cytochrome P450,2C70	226105	−2.75	−4.22	−1.23	−1.68*	−1.05	−1.05	1.04
CYP2C65	cytochrome P450,2C65	72303	1.44	1.63	−1.93	1.98	46.78	2.28	8.63**
CYP2D10	cytochrome P450,2D10	13101	−1.47	−1.09	−1.02	−1.03	1.33	−1.00	1.02
CYP2D26	cytochrome P450,2D26	76279	−1.17	−1.21	1.06	−1.01	−1.12	−1.03	−1.08
CYP3A11	cytochrome P450,3A11	13112	−1.23	1.40	1.03	1.06	4.61	1.12	1.20
CYP3A41A	cytochrome P450,3A41A	53973	−2.08	1.11	1.24	1.58*	2.01	1.39	1.25
CYP3A25	cytochrome P450,3A25	56388	−1.94	−1.70	1.01	−1.01	1.04	1.13	1.12
CYP3A13	cytochrome P450,3A13	13113	−1.54	1.19	1.22	1.38*	1.52	1.75	1.62**
EPHX1	epoxide hydrolase 1, microsomal	13849	1.22	1.78	1.16	1.60*	1.82	1.33	1.59*
EPHX2	epoxide hydrolase 2, cytoplasmic	13850	2.25	2.34	1.45	1.67**	1.04	1.05	1.07
GSTA3	glutathione S-transferase A3	14859	1.08	−1.04	1.05	1.26	1.11	1.11	1.13
GSTA4	glutathione S-transferase A4	14860	−2.01	−1.10	−1.02	1.52	1.37	−1.20	1.36
GSTA5	glutathione S-transferase A5	14857	−1.12	1.44	1.19	2.76*	2.26	1.15	2.13
GSTK1	glutathione S-transferase kappa 1	76263	1.85	1.43	1.02	−1.04	−1.30	−1.26	−1.27
GSTM1	glutathione S-transferase M1	14863	−2.12	−1.56	−1.51	1.77	2.54	1.18	1.97
GSTM3	glutathione S-transferase, mu 3	14864	−1.32	1.50	1.16	2.44*	1.83	1.57	2.59*
GSTM4	glutathione S-transferase M4	14865	2.07	3.13	1.30	2.40*	2.48	1.40	2.63*
GSTP1	glutathione S-transferase pi 1	14870	−2.79	4.14	−1.16	1.00	2.87	−1.06	−1.03
GSTT2	glutathione S-transferase theta 2	14872	1.64	2.74	1.42	1.83**	1.13	1.16	1.43**
GSTT3	glutathione S-transferase, theta 3	103140	2.10	1.13	1.41	1.61	1.77	1.30	1.85**
GSTZ1	glutathione transferase zeta 1	14874	−1.36	−1.14	−1.03	−1.08	1.01	1.03	1.01
MGST1	microsomal glutathione S-transferase 1	56615	1.28	1.24	−1.02	1.01	1.21	1.04	1.01
MGST3	microsomal glutathione S-transferase 3	66447	1.73	1.60	1.24	1.80*	−1.54	−1.31	−1.06
POR	P450 (cytochrome) oxidoreductase	18984	−1.26	2.63	1.27	1.94	2.04	2.91	3.30**
UGT2B17	UDP glucuronosyltransferase 2B17	71773	−3.90	−1.13	−1.03	1.02	1.24	1.03	−1.01
UGT2B4	UDP glucuronosyltransferase 2B4	552899	−1.37	−1.93	−1.26	−1.23*	1.35	1.01	1.03
UGT2B7	UDP glucuronosyltransferase 2B7	231396	−1.19	−1.20	−1.05	−1.05	1.16	1.04	−1.00

^1^From Rosen et al. (2008),

*Significantly different than control (*P* ≤ .03),

**Significantly different than control (*P* ≤ .0025).

**Table 8:** Average fold change for genes related to cholesterol biosynthesis in wild-type and PPAR*α*-null male mice following a seven-day exposure to Wy-14,643^1^, PFOA^1^, or PFOS.

					WT			Null	
Symbol	Gene name	Entrez no.	Wy14,643 50 mg/kg	PFOA 3 mg/kg	PFOS 3 mg/kg	PFOS 10 mg/kg	PFOA 3 mg/kg	PFOS 3 mg/kg	PFOS 10 mg/kg
CYP51	cytochrome P450, family 51	13121	2.85	1.37	1.27	2.10*	1.37	2.99	1.93**
FDFT1	farnesyl-diphosphate farnesyltransferase 1	14137	2.30	1.28	1.29	1.73*	1.09	2.00	1.92**
FDPS	farnesyl diphosphate synthase	110196	3.19	1.79	1.16	1.38	1.83	1.84	1.96**
HMGCR	3-hydroxy-3-methylglutaryl -CoA reductase	15357	1.79	−1.08	1.19	1.97**	1.20	1.85	1.80*
HMGCS1	3-hydroxy-3-methylglutaryl -CoA synthase 1	208715	6.67	1.79	1.15	1.61	−1.06	3.11	1.86*
HMGCS2	3-hydroxy-3-methylglutaryl -CoA synthase 2	15360	1.17	1.54	1.28	1.34*	1.25	−1.08	−1.28*
IDI1	isopentenyl-diphosphate delta isomerase 1	319554	3.14	1.61	1.35	1.62	1.40	1.96	1.57*
LSS	lanosterol synthase	16987	1.73	1.08	1.12	1.41	−1.26	1.98	2.13**
MVK	mevalonate kinase	17855	1.45	−1.24	1.12	1.22	−1.02	1.57	1.52**
PMVK	phosphomevalonate kinase	68603	3.23	2.04	1.36	1.51*	1.20	1.58	1.53**
SQLE	squalene epoxidase	20775	3.10	1.05	1.17	1.46	1.26	2.25	1.98**

^1^From Rosen et al. (2008), *Significantly different than control (*P* ≤ .03),

**Significantly different than control (*P* ≤ .0025).

**Table 9:** Average fold change for genes related to oxidative phosphorylation/electron transport in wild-type and PPAR*α*-null male mice following a seven-day exposure to Wy-14,643^1^, PFOA^1^, or PFOS.

					WT			Null	
Symbol	Gene name	Entrez no.	Wy14,643 50 mg/kg	PFOA 3 mg/kg	PFOS 3 mg/kg	PFOS 10 mg/kg	PFOA 3 mg/kg	PFOS 3 mg/kg	PFOS 10 mg/kg
ATP5D	ATP synthase H+ transporting, F1delta	66043	1.03	1.10	1.04	1.09	−1.17	−1.22	−1.13*
ATP5E	ATP synthase H+ transporting, F1epsilon	67126	−1.10	1.21	−1.00	1.03	−1.17	−1.32	−1.38**
ATP5G2	ATP synthase H+ transporting, F0, C2	67942	−1.09	−1.03	1.10	−1.10	−1.10	−1.33	−1.26**
ATP5G3	ATP synthase H+ transporting, F0, C3	228033	1.62	1.48	−1.01	1.05	−1.10	−1.12	−1.10**
ATP5H	ATP synthase H+ transporting, F0, D	71679	1.18	1.10	1.05	1.06	−1.01	−1.30	−1.38**
ATP5I	ATP synthase H+ transporting, F0, E	11958	−1.01	−1.45	−1.03	1.10	1.17	−1.38	−1.50**
ATP5J	ATP synthase H+ transporting, F0, F6	11957	−1.20	1.44	−1.04	−1.07	−1.14	−1.25	−1.35**
ATP5J2	ATP synthase H+ transporting,F0, F2	57423	2.38	−1.56	−1.05	−1.09	1.03	−1.29	−1.35**
ATP5L	ATP synthase H+ transporting, F0, G	27425	1.58	1.21	−1.02	1.00	−1.05	−1.33	−1.30**
ATP5O	ATP synthase H+ transporting, F1, O	28080	1.12	1.16	1.06	1.22	−1.03	−1.33	−1.31**
ATP6V0B	ATPase, H+ transporting, V0 unit b	114143	−1.37	−1.25	1.03	−1.09	1.05	−1.22	−1.20**
ATP6V1F	ATPase, H+ transporting, V1 unit F	66144	−1.18	1.23	1.00	1.05	1.01	−1.33	−1.28**
COX4I1	cytochrome c oxidase unit IV isoform 1	12857	1.14	1.15	1.02	1.03	−1.15	−1.19	−1.16**
COX5A	cytochrome c oxidase unit Va	12858	1.25	1.12	−1.02	1.09	−1.13	−1.26	−1.33**
COX5B	cytochrome c oxidase unit Vb	12859	1.19	1.33	1.09	1.08	−1.27	−1.27	−1.35**
COX6B1	cytochrome c oxidase unit VIb1	110323	1.32	1.39	−1.01	1.10*	−1.12	−1.25	−1.19*
COX6C	cytochrome c oxidase unit VIc	12864	1.62	−1.23	1.03	−1.05	1.21	−1.22	−1.25**
COX7A2	cytochrome c oxidase unit VIIa 2	12866	−1.68	−1.08	−1.04	−1.04	−1.57	−1.39	−1.37**
COX7C	cytochrome c oxidase unit VIIc	12867	1.22	1.32	−1.03	−1.28*	−1.05	−1.23	−1.19**
COX8A	cytochrome c oxidase unit 8A	12868	1.34	1.34	1.02	1.04	1.07	−1.23	−1.13*
NDUFA1	NADH dehydrogenase 1 alpha1	54405	−1.19	1.13	−1.03	−1.11	−1.25	−1.31	−1.49**
NDUFA2	NADH dehydrogenase 1 alpha 2	17991	1.06	1.18	1.04	1.04	−1.06	−1.26	−1.33**
NDUFA3	NADH dehydrogenase 1 alpha 3	66091	1.60	1.60	1.06	1.16*	−1.06	−1.37	−1.30**
NDUFA4	NADH dehydrogenase 1 alpha 4	17992	1.02	2.46	−1.00	1.01	3.16	−1.12	−1.11**
NDUFA5	NADH dehydrogenase 1 alpha 5	68202	1.41	1.26	1.10	1.11	−1.07	−1.55	−1.73**
NDUFA6	NADH dehydrogenase 1 alpha 6	67130	1.10	1.06	1.02	−1.04	−1.02	−1.34	−1.29**
NDUFA7	NADH dehydrogenase 1 alpha 7	66416	−1.14	−1.01	1.09	1.12	−1.17	−1.45	−1.38**
NDUFA8	NADH dehydrogenase 1 alpha 8	68375	1.14	1.33	1.00	1.09	1.05	−1.29	−1.18*
NDUFA12	NADH dehydrogenase 1 alpha12	66414	1.47	1.16	−1.03	1.06	1.06	−1.51	−1.40**
NDUFA13	NADH dehydrogenase 1 alpha13	67184	−1.12	−1.16	−1.03	−1.03	−1.08	−1.26	−1.28**
NDUFA9	NADH dehydrogenase 1 alpha 9	66108	1.18	1.07	1.02	−1.01	−1.09	−1.20	−1.19**
NDUFAB1	NADH dehydrogenase 1, alpha/beta 1	70316	1.56	1.19	1.05	1.23*	−1.07	−1.31	−1.44*
NDUFB2	NADH dehydrogenase 1 beta 2	68198	−2.31	−3.32	1.04	1.11	1.49	−1.31	−1.35**
NDUFB3	NADH dehydrogenase 1 beta 3	66495	1.55	1.93	1.09	1.19	1.05	−1.41	−1.32**
NDUFB4	NADH dehydrogenase 1 beta 4	68194	−1.03	1.17	−1.01	1.06	−1.13	−1.45	−1.46**
NDUFB5	NADH dehydrogenase 1 beta 5	66046	1.21	1.13	1.08	1.03	1.05	−1.28	−1.41**
NDUFB6	NADH dehydrogenase 1 beta 6,	230075	1.32	−1.03	1.04	1.19	−1.02	−1.38	−1.36**
NDUFB7	NADH dehydrogenase 1 beta 7,	66916	1.02	1.14	1.04	1.11	−1.11	−1.40	−1.29**
NDUFB9	NADH dehydrogenase 1 beta 9,	66218	1.19	1.01	1.05	1.01	−1.08	−1.22	−1.25**
NDUFB11	NADH dehydrogenase 1 beta 11	104130	−1.29	1.05	1.05	1.06	−1.00	−1.26	−1.23**
NDUFC1	NADH dehydrogenase 1 unknown 1	66377	−1.28	1.84	1.07	1.21*	1.17	−1.28	−1.37**
NDUFC2	NADH dehydrogenase 1 unknown, 2	68197	−1.02	1.13	1.06	1.06	−1.13	−1.37	−1.33**
NDUFS4	NADH dehydrogenase Fe-S protein 4	17993	1.51	1.21	1.12	−1.12	1.07	−1.41	−1.40**
NDUFS5	NADH dehydrogenase Fe-S protein 5	595136	1.16	1.13	−1.01	1.08	1.02	−1.37	−1.44**
NDUFS7	NADH dehydrogenase Fe-S protein 7	75406	1.09	1.40	1.09	1.13*	1.07	−1.28	−1.15
NDUFS6	NADH dehydrogenase Fe-S protein 6	407785	−1.32	1.06	−1.01	1.02	−1.14	−1.30	−1.32**
NDUFV2	NADH dehydrogenase flavoprotein 2	72900	1.38	1.09	1.06	1.07	−1.02	−1.24	−1.24**
NDUFV3	NADH dehydrogenase flavoprotein 3,	78330	1.12	1.16	−1.03	−1.01	−1.14	−1.35	−1.39**
UCRC	ubiquinol-cytochrome c reductase	66152	1.58	1.26	1.10	1.27	1.07	−1.40	−1.27**
UHRF1BP1	UHRF1 binding protein 1	224648	−1.03	1.36	−1.08	1.06	1.15	1.23	1.15**
UQCR	ubiquinol-cytochrome c reductase	66594	1.26	1.40	1.04	1.14*	1.09	−1.28	−1.19*
UQCRC2	ubiquinol-cytochrome c reductase CP II	67003	1.09	1.17	1.07	1.13	−1.04	−1.11	−1.27*
UQCRQ	ubiquinol-cytochrome c reductase 3 unit 7	22272	1.01	1.08	1.07	1.12*	−1.07	−1.18	−1.21**

^1^From Rosen et al. [1], *Significantly different than control (*P* ≤ .03),**Significantly different than control (*P* ≤ .0025).

**Table 10:** Average fold change for genes related to ribosome biogenesis following a seven-day exposure to Wy-14,643^1^, PFOA^1^, or PFOS in wild-type and PPAR*α*-null male mice.

					WT			Null	
Symbol	Gene name	Entrez no.	Wy14,643 50 mg/kg	PFOA 3 mg/kg	PFOS 3 mg/kg	PFOS 10 mg/kg	PFOA 3 mg/kg	PFOS 3 mg/kg	PFOS 10 mg/kg
MRPL12	mitochondrial ribosomal protein L12	56282	−1.16	1.25	1.07	1.14*	−1.16	−1.18	−1.12*
MRPL13	mitochondrial ribosomal protein L13	68537	1.32	1.33	1.12	1.35*	1.01	−1.21	−1.42**
MRPL17	mitochondrial ribosomal protein L17	27397	1.68	1.76	1.10	1.43**	1.13	−1.13	1.09
MRPL23	mitochondrial ribosomal protein L23	19935	−1.14	−1.04	−1.00	1.10	1.09	−1.38	−1.20*
MRPL33	mitochondrial ribosomal protein L33	66845	1.22	1.26	1.07	1.05	1.04	−1.29	−1.28**
MRPS12	mitochondrial ribosomal protein S12	24030	−1.24	1.18	1.05	1.12	1.02	−1.27	−1.15
MRPS18A	mitochondrial ribosomal protein S18A	68565	−1.46	1.34	1.04	1.28*	1.60	−1.19	−1.06
RPL10	ribosomal protein L10	110954	−1.15	−1.21	1.02	1.03	1.07	−1.10	−1.02
RPL10A	ribosomal protein L10A	19896	−1.11	1.10	1.03	1.05	1.00	−1.07	1.01
RPL11	ribosomal protein L11	67025	1.14	1.12	1.10	1.11*	1.15	−1.15	−1.09
RPL12	ribosomal protein L12	269261	1.01	1.37	1.08	1.15*	1.11	−1.08	1.05
RPL13A	ribosomal protein L13a	22121	−1.14	1.03	1.07	1.12*	−1.17	−1.15	−1.10
RPL14	ribosomal protein L14	67115	−1.28	−1.06	1.15	1.23**	−1.13	−1.18	−1.22*
RPL17	ribosomal protein L17	319195	−1.27	1.15	1.03	1.12	−1.52	−1.10	−1.09
RPL18	ribosomal protein L18	19899	−1.11	1.28	1.04	1.07*	1.19	−1.27	−1.09*
RPL18A	ribosomal protein L18a	76808	1.65	−1.37	1.04	1.11*	1.08	−1.15	−1.02
RPL19	ribosomal protein L19	19921	1.22	1.23	1.01	1.05	1.07	−1.11	−1.03
RPL21	ribosomal protein L21	19933	2.00	1.55	1.03	1.09	1.18	−1.20	−1.18
RPL22	ribosomal protein L22	19934	1.17	1.45	1.06	1.29**	1.08	−1.25	−1.14*
RPL23	ribosomal protein L23	65019	−1.07	1.35	1.06	1.06	1.22	−1.24	−1.16
RPL24	ribosomal protein L24	68193	−1.13	1.07	1.06	1.09*	−1.00	−1.19	−1.11*
RPL26	ribosomal protein L26	19941	1.04	1.22	1.03	1.03	1.07	−1.22	−1.18**
RPL27	ribosomal protein L27	19942	1.04	−1.01	1.08	1.38**	1.06	−1.25	−1.40*
RPL27A	ribosomal protein L27a	26451	−1.07	1.07	−1.00	1.17	1.26	−1.17	−1.09
RPL28	ribosomal protein L28	19943	1.29	1.04	1.01	1.11*	1.67	−1.22	−1.10
RPL29	ribosomal protein L29	19944	1.16	−1.30	1.04	1.09	1.08	−1.23	−1.17
RPL3	ribosomal protein L3	27367	−1.00	−1.14	1.01	1.09	−1.01	−1.03	1.06
RPL30	ribosomal protein L30	19946	−1.15	−1.07	1.02	−1.21	−1.04	−1.29	−1.23**
RPL31	ribosomal protein L31	114641	1.11	1.37	1.09	1.05	1.29	−1.18	−1.12*
RPL32	ribosomal protein L32	19951	1.06	1.11	1.02	1.12*	1.08	−1.16	−1.03
RPL34	ribosomal protein L34	68436	−1.26	1.16	−1.07	1.05	−1.04	−1.22	−1.31**
RPL35	ribosomal protein L35	66489	−1.03	1.15	1.13	1.26**	1.04	−1.17	−1.11
RPL36	ribosomal protein L36	54217	−1.07	1.12	1.09	1.23*	1.07	−1.27	−1.20*
RPL37	ribosomal protein L37	67281	−1.16	−1.18	1.04	1.27*	1.17	−1.19	−1.10**
RPL37A	ribosomal protein L37a	19981	−1.15	−1.09	1.03	1.16	−1.12	−1.22	−1.19*
RPL38	ribosomal protein L38	67671	−1.17	1.14	−1.01	1.06	−1.03	−1.18	−1.10
RPL39	ribosomal protein L39	67248	1.04	1.02	1.06	1.13*	1.07	−1.18	−1.16**
RPL4	ribosomal protein L4	67891	1.16	1.43	1.03	1.03	1.32	1.03	1.04
RPL41	ribosomal protein L41	67945	−1.06	1.14	1.05	1.06	−1.13	−1.20	−1.26*
RPL5	ribosomal protein L5	19983	−1.21	1.02	1.24	1.09*	−1.05	−1.05	−1.11
RPL6	ribosomal protein L6	19988	1.01	−1.08	1.00	1.05	1.15	−1.05	1.03
RPL7A	ribosomal protein L7a	27176	−1.02	−1.11	1.01	1.01	−1.02	−1.07	1.01
RPL9	ribosomal protein L9	20005	−1.35	−1.08	1.03	1.07	−1.11	−1.19	−1.12*
RPS10	ribosomal protein S10	67097	−1.02	1.02	1.05	1.07	1.00	−1.17	−1.12*
RPS11	ribosomal protein S11	27207	1.05	−1.74	−1.01	1.11	1.06	−1.24	−1.14*
RPS12	ribosomal protein S12	20042	1.16	1.22	1.11	1.19	1.22	−1.21	−1.12
RPS13	ribosomal protein S13	68052	−1.03	1.10	1.07	1.22*	1.11	−1.27	−1.22*
RPS14	ribosomal protein S14	20044	−1.03	1.19	1.05	1.11*	1.01	−1.17	−1.11**
RPS15A	ribosomal protein S15a	267019	−1.05	1.05	1.02	1.12	1.02	−1.14	−1.20
RPS16	ribosomal protein S16	20055	−1.09	1.05	1.05	1.07	−1.02	−1.12	−1.07
RPS17	ribosomal protein S17	20068	1.00	1.16	1.04	−1.19*	1.01	−1.19	−1.15*
RPS19	ribosomal protein S19	20085	−1.07	1.23	1.08	1.19**	−1.00	−1.14	−1.05
RPS2	ribosomal protein S2	16898	−1.09	1.02	1.04	1.02	−1.16	−1.03	1.04
RPS20	ribosomal protein S20	67427	−1.40	1.21	1.04	1.15	1.25	−1.11	−1.13
RPS21	ribosomal protein S21	66481	1.11	−1.32	1.15	1.38	1.39	−1.32	−1.25**
RPS23	ribosomal protein S23	66475	1.01	1.04	−1.00	1.04	1.09	−1.21	−1.10*
RPS24	ribosomal protein S24	20088	1.58	1.62	1.11	−1.29*	1.75	−1.16	−1.19**
RPS25	ribosomal protein S25	75617	−1.23	1.01	1.09	1.13*	−1.02	−1.30	−1.17*
RPS26	ribosomal protein S26	27370	1.32	1.30	1.04	1.16*	1.14	−1.20	−1.08
RPS27A	ribosomal protein S27a	78294	1.05	−1.05	−1.00	1.02	1.09	−1.08	−1.05
RPS27L	ribosomal protein S27-like	67941	1.72	1.28	1.07	1.14*	1.19	−1.18	−1.17*
RPS28	ribosomal protein S28	54127	−1.19	−1.03	1.03	1.06	−1.05	−1.28	−1.17*
RPS29	ribosomal protein S29	20090	−1.26	−1.05	−1.02	1.01	−1.03	−1.19	−1.20**
RPS3	ribosomal protein S3	27050	−1.04	1.29	1.03	1.20*	−2.88	−1.11	−1.06
RPS3A	ribosomal protein S3A	544977	−1.18	−1.07	1.02	−1.01	−1.05	−1.10	−1.03
RPS5	ribosomal protein S5	20103	−1.16	1.18	1.06	1.09*	−1.02	−1.13	−1.00
RPS6	ribosomal protein S6	20104	−1.20	−1.02	−1.20	1.06	−1.02	−1.14	−1.06*
RPS8	ribosomal protein S8	20116	1.19	−1.05	1.07	1.13*	1.04	−1.29	−1.13
RPS9	ribosomal protein S9	76846	−1.39	1.30	1.05	1.07	1.05	−1.08	−1.04

^1^From Rosen et al. (2008), *Significantly different than control (*P* ≤ .03),

**Significantly different from control (*P* ≤ .0025).
